# A novel therapeutic approach for endometriosis using adipose-derived stem cell-derived conditioned medium- A new hope for endometriotic patients in improving fertility

**DOI:** 10.3389/fendo.2023.1158527

**Published:** 2023-05-24

**Authors:** S. Joseph Huang, Chun-Yen Huang, Yu-Hao Huang, Jai-Hong Cheng, Ya-Chun Yu, Jui-Chi Lai, Yi-Pei Hung, Chi-Chang Chang, Li-Yen Shiu

**Affiliations:** ^1^ Department of Obstetrics and Gynecology, E-Da Hospital, I-Shou University, Kaohsiung, Taiwan; ^2^ School of Medicine, College of Medicine, I-Shou University, Kaohsiung, Taiwan; ^3^ Department of Obstetrics and Gynecology, University of South Florida, Tampa, FL, United States; ^4^ Department of Plastic Surgery, E-Da Dachang Hospital, I-Shou University, Kaohsiung, Taiwan; ^5^ Center for Shockwave Medicine and Tissue Engineering, Kaohsiung Chang Gung Memorial Hospital and College of Medicine, Chang Gung University, Kaohsiung, Taiwan; ^6^ Department of Medical Research, Kaohsiung Chang Gung Memorial Hospital and College of Medicine, Chang Gung University, Kaohsiung, Taiwan; ^7^ Cell Therapy Center, E-Da Hospital, I-Shou University, Kaohsiung, Taiwan; ^8^ UnicoCell Biomed, Taipei, Taiwan; ^9^ Department of Obstetrics and Gynecology, E-Da Dachang Hospital, I-Shou University, Kaohsiung, Taiwan

**Keywords:** endometriosis, adipose-derived stem cell, conditioned medium, proteomics, PTX3

## Abstract

**Introduction:**

Endometriosis is defined as the growth of endometrial glands and stromal cells in a heterotopic location with immune dysregulation. It usually leads to chronic pelvic pain and subfertility. Although various treatments are available, the recurrence rate remains high. Adipose tissue is an abundant source of multipotent mesenchymal adipose-derived stem cells (ADSCs). ADSCs display effects on not only tissue regeneration, but also immune regulation. Thus, the current study aims to test the effects of ADSCs on the growth of endometriosis.

**Methods:**

ADSCs isolated from lipoaspiration-generated adipose tissue and their conditioned medium (ADSC-CM) were subjected to quality validation, including karyotyping as well as growth promotion and sterility tests for microbial contamination under Good Tissue Practice and Good Manufacturing Practice regulations. An autologous endometriosis mouse model was established by suturing endometrial tissue to peritoneal wall followed by treating with DMEM/F12 medium, ADSC-CM, ADSCs or ADSC-CM+ADSCs for 28 days. The area of endometriotic cysts and the degree of pelvic adhesion were measured. ICAM-1, VEGF and caspase 3 expression was assessed by quantitative reverse transcription polymerase chain reaction (qRT-PCR) and immunohistochemistry. Moreover, the mice were allowed to mate and deliver. The pregnancy outcomes were recorded. The ADSC-CM was subjected to proteomics analysis with further data mining with Ingenuity Pathway Analysis (IPA).

**Results:**

Both ADSC-CM and ADSCs passed quality validation. ADSC-CM reduced the area of endometriotic cysts. The inhibition by ADSC-CM was obliterated by adding ADSCs. The presence of ADSCs with or without ADSC-CM increased the peritoneal adhesion. ADSC-CM inhibited ICAM-1 and VEGF mRNA and protein expression, whereas the addition of ADSCs not only did not inhibit by itself, but also blocked the inhibition by ADSC-CM. The resorption rate was reduced by ADSC-CM. The number of live birth/dam and the survival rate of pup at 1 week-old were both increased by ADSC-CM in mice with endometriosis. IPA demonstrated that PTX3 was potentially critical for the inhibition of endometriosis by ADSC-CM due to its anti-inflammatory and antiangiogenic properties as well as its importance in implantation.

**Conclusion:**

ADSC-CM inhibited endometriosis development and improved pregnancy outcomes in mice. Potential translation to clinical treatment for human endometriosis is expected.

## Introduction

Endometriosis is initially a benign but frequently progressive disease that occurs in 10% of reproductive age women, 60% of women with pelvic pain, and 30% to 50% of infertile women. It is associated with a wide spectrum of clinical sequelae, including secondary dysmenorrhea and/or dyspareunia, infertility as well as symptoms associated with gastrointestinal and urinary tract involvement (1). Although coelomic metaplasia and direct transplantation *via* lymphatic or vascular metastasis may play a role, the etiology of endometriosis is frequently attributed to retrograde menstruation. Since only a small percentage of women with retrograde menstruation develop endometriosis, additional factors are likely to play roles in implantation, growth, and angiogenesis of ectopic endometrial tissue. Moreover, changes in the immune system have been recently suggested to play a causative role in the pathogenesis of endometriosis (2, 3). Despite evidence of abnormalities in cell-mediated and humoral immune response in both peripheral blood and peritoneal fluid of patients with endometriosis (4–7), the cause of these aberrant immune responses remains unclear.

Factors to be considered in the management of endometriosis include the age and reproductive desires of the patient, the stage of the disease as well as, most importantly, the symptoms. Surgical treatment is considered appropriate, especially for advanced stages of the disease. Specifically, conservative surgery preserves the reproductive organs and is an effective treatment for endometriosis-associated pain. Laparoscopic surgery is an effective approach with the goal of eradicating visible endometriotic lesions. Hysterectomy with bilateral salpingo-oophorectomy remains a main therapy for refractory endometriosis-associated pain in patients who have completed childbearing.

Endometriosis is also responsive to hormonal therapy. A hypoestrogenic and anovulatory state induced by various hormonal drugs leads to atrophic change of endometriotic tissue. The use of hormonal therapies, including oral contraceptives, progestins, danazol or such GnRH agonists as leuprolide acetate, goserelin acetate, selective progesterone receptor modulators, selective estrogen receptor modulators, and nafarelin acetate, is commonly accompanied with non-steroid anti-inflammatory drugs.(8) Moreover, RU486 (mifepristone), GnRH antagonists, pentoxifylline, tumor necrosis factor-alpha (TNF-α) inhibitors, matrix metalloproteinases, and angiogenesis inhibitors are still under investigation (8). However, clinical observations show that the outcomes of medical treatments are unpredictable. Although both surgical and medical treatments have been shown to be effective for treating complications associated with endometriosis, the recurrent rate is estimated to be more than 20% 2 years and 40-50% 5 years after treatment(s). Thus, the development of new therapeutic strategies is required to improve the efficacy of treatment for endometriosis.

Adipose-derived stem cells (ADSCs) isolated from adipose tissue are multipotent mesenchymal stem cells (MSCs) (9). Human adipose tissue is ubiquitous and easily obtainable in large quantity under local anesthesia with minimal patient discomfort. Thus, ADSCs can serve as an alternative source of stem cells for mesenchymal tissue-based regeneration and engineering. Recently, ADSC therapy has been utilized to repair various organs (10–12). For renal fibrosis, ADSCs were found to regulate inflammatory responses and reduce epithelial–mesenchymal transition both early and late after injury (13). Furthermore, ADSC-conditioned medium (ADSC-CM) exhibits anti-oxidative (14), anti-proliferative (15), immunoregulatory, and wound-healing effects on various organ systems (16).

MSC-CM contains adipokines, cytokines, growth factors, and extracellular vesicles (17–20). The complexity of ADSCs and ADSC-CM mandate further investigation of their potential therapeutic applications and their underlying mechanisms (21). Thus, this study aims to examine the effects of human ADSC-CM on the development of inflammation-associated endometriosis using a mouse model. The findings can potentially be translated to clinical application to combat endometriosis.

## Materials and methods

### Human ADSCs isolation and culture

Before processing, all chemical reagents, supplements, buffer solutions and culture media were checked for microbial contamination. The procedures of sterility test followed Chinese Pharmacopoeia 8^th^ edition, general chapter <7001>. All isolation and cultivation procedures were accomplished in a clean laboratory at E-Da Cell Therapy Center and under Good Tissue Practice (GTP) and Good Manufacturing Practice (GMP) regulations.

The human adipose tissues were obtained from lipoaspiration under E-Da Hospital IRB (EMRP04109N) approval. As previously described, (22) after washing with DPBS x2, the tissues were minced and digested with 0.1% collagenase I (GMP-grade Clzyme AS, VitaCyte, Indianapolis, IN, USA) in DMEM for 1h at 37°C with gentle agitation. Then, the digestate was centrifuged for 10min at 1,000 rpm. The cell pellet was re-suspended in DMEM/F12 supplemented with 5% platelet-rich plasma (PRP, UltraGRO™-PURE GI, AventaCell BioMedical, USA) and filtered through a 100-μm mesh filter to remove debris. The filtrate was centrifuged at 400× g for 10min at room temperature followed by re-suspension in 10 mL of ammonium-chloride-potassium (ACK) lysis buffer. After adding 10 mL of 1× DPBS buffer containing 1% antibiotics (20 μg/mL penicillin/streptomycin), the mixture was centrifuged at 400 ×g for 5min followed by re-suspension in 5 mL of complete medium (DMEM/F12 containing 5% PRP and 1% antibiotics) and cultured at 37°C in 5% CO_2_. After removal of unattached cells at the third day of cultivation, the complete medium was replaced every 3-4 days until the culture reached 85-90% confluence and passaged. Chromosomal stability of ADSCs was checked at passage three by karyotyping.

### ADSC characterization

The expression of CD14 (for monocytes), CD19 (for B-cells), CD29 (for ADSCs), CD31 (for endothelial cells), CD34 (for hematopoietic cells), CD44 (for ADSCs), CD45 (pan-leukocyte marker), CD73 (for ADSCs), CD90 (for ADSCs) and CD105 (for ADSCs) in the third-passage cells were examined by flow cytometric analysis. After fixation with ice-cold methanol, 100 μL of cell suspension with density at 1 × 10^6^ cells/mL were stained with each specific antibody for 30min at 37°C in dark. The samples were centrifuged for 5min at 400× g, washed with DPBS twice, and re-suspended in 0.4 mL of DPBS before analyzed by a BD Accuri flow cytometer (BD, Franklin Lakes, NJ, USA) and FlowJo v.10.6.1 software (BD Bioscience).

### Conditioned medium production and quality control

Human ADSCs at the third passage were seeded to T75 flasks at a density of 2 × 10^4^ cells/cm^2^. At 80-90% confluence, ADSCs were washed with 1× DPBS and cultured with 10 mL of serum-free DMEM/F12 for 48h at 37°C in 5% CO_2_. Then, CM was collected and filtered through a 0.22-μm filter to remove cell debris. CM was aliquoted and stored at -80°C for future experiments.

### CM safety test

In support of pharmaceutical quality for media used in a GMP facility, culture medium for ADSCs and ADSC-CM were subjected to growth promotion test using such inoculum as Staphylococcus aureus, Pseudomonas aeruginosa, Bacillus subtilis, Candida albicans or Aspergillus niger, prior to sterility test. Before releasing CM from Cell Therapy Center, its safety was confirmed by various tests for sterility, bacterial endotoxin and mycoplasma. All safety tests were performed under the guidance of Chinese Pharmacopoeia 8^th^ edition, general chapters <7001>, <7008> and <7009>. Direct transfer sterility test in tryptic soy broth (TSB) and fluid thioglycollate medium (FTM) verified the sterility of CM by incubating for 14 days with daily examination for the presence of microbials. Mycoplasma was checked by the nucleic acid amplification test (NAAT) based on Chinese Pharmacopoeia 8^th^ edition, general chapter <7009>. Limulus Amebocyte Lysate (LAL) test used chromogenic methods to measure the concentration of bacterial endotoxin in CM, which is expected to be less than 0.5 EU/mL.

### Endometriosis mouse model

The animal studies were performed under E-Da Hospital Institutional Animal Care and Use Committee (IACUC-EDAH-108040) approval. Nine-week-old female C57BL/6JNarl mice purchased from BioLASCO Taiwan Co., Ltd. were kept under controlled conditions (24˚C, 12:12 light-dark cycle with lights on at 6:00 AM) and anesthetized with Zoteil (VIRBAC, Pukete, Hamilton, France) by intraperitoneal injection. The uterus was ligated at the internal cervical os followed by removal of uterus with both ovaries spared. Both uterine horns were longitudinally opened. Four pieces of uterine tissues with identical size generated by a disposable 2-mm dermal biopsy punch (Life Technologies, Carlsbad, CA, USA) were sutured to the peritoneal wall using a 6-0 monofilament nylon (UNIK, New Taipei City, Taiwan) with two pieces on each side (23). Subsequently, the mice were intraperitoneally treated with medium (control) (1 mL), ADSC-CM (1 mL), ADSC (1 x 10^6^ cells) or ADSC-CM (1 mL) + ADSC (1 x 10^6^ cells) only once before suturing the abdominal wound and sacrificed at day 28 to harvest the endometriotic tissues.

**Figure 1:**
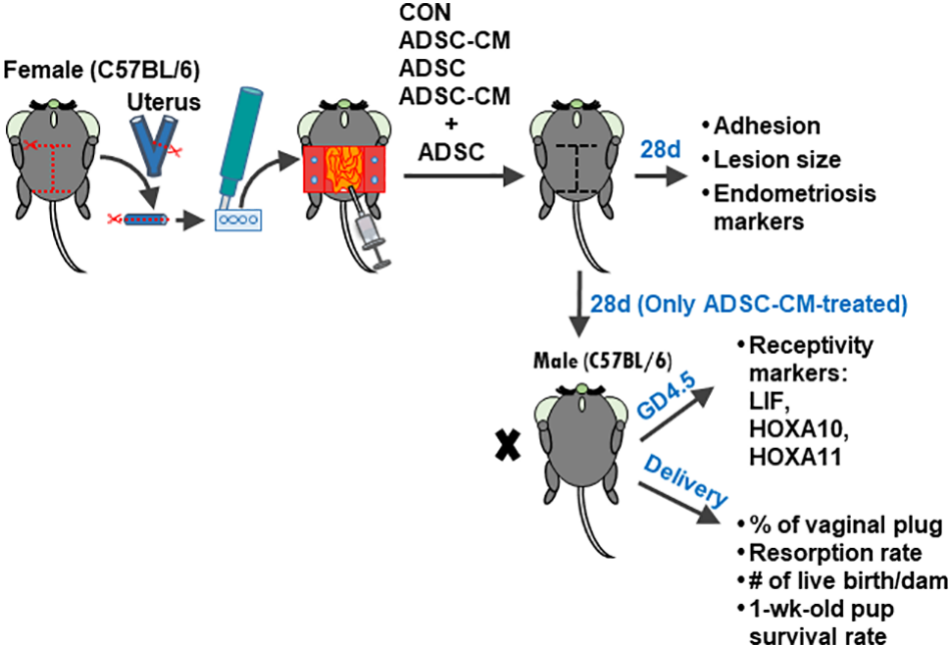
Experimental design Four pieces of uterine tissue were generated from the uterus of a female C57BL/6JNarl mice using a disposable 2-mm dermal biopsy punch and sutured to the peritoneal wall with two pieces on each side. Then, the culture medium, ADSC, ADSC-CM or ADSC + ADSC-CM was added into the peritoneal cavity. The endometriotic lesions were harvested at 28 days. To examine the pregnancy outcomes, the mouse was mated with a male C57BL/6JNarl at day 28 and allowed to deliver at term. The percentage of vaginal plug, resorption rate, live births per dam, and pup survival rate at 1 week-old were recorded.

A total of 6 batches of ADSCs and ADSC-CM were used in 6 independent experiments with triplicate in each experiment. One half of the tissues were fixed in 4% paraformaldehyde for 24h at 4°C and the other half were snap-frozen and stored in −80°C for future use. The formation of adhesion bands between peritoneum and endometriotic tissues was graded based on a 0 to 3 scale: 0: no adhesion. 1: mild adhesions that can be easily separated. 2: moderate adhesions that can be separated by a pair of forceps. 3: dense adhesions that require to use scissors to separate. The area of the lesion was measured using Image-J v1.53 DIA software (IHC Image Analysis Toolbox, National Institutes of Health, Washington, USA; https://imagej.nih.gov/ij/index.html). Since only ADSC-CM was effective in reducing the lesion size, the treatments of ADSC and ADSC-CM + ADSC were excluded in further studies of eutopic endometrial receptivity and pregnancy outcomes. For pregnancy outcomes, the female mice after 28-day treatment with either medium (control) (6 mice) or ADSC-CM (8 mice) were mated with an 8-week-old male C57BL/6JNarl and allowed to deliver at term. The endometrial receptivity of the eutopic endometrium were examined at gestational day (GD) 4. The pregnancy outcomes, including: 1) number of vaginal plug; 2) resorption rate; 3) live birth per dam; 4) pup survival rate at 1 week-old, were recorded (**Figure 1**).

### H & E stain

Paraffin-embedded tissue sections (4 μm) prepared using an SM200R microtome (Leica, Wetzlar, Germany) were placed on slides pre-coated with poly-L-lysine followed by deparaffinization and rehydration. The sections were stained with hematoxylin and eosin followed by clearing with xylene for 5min x2 before mounting with DPX mounting medium and examination under an Olympus BX43 light microscope.

### Quantitative reverse transcription-polymerase chain reaction

Total RNA was extracted from the endometriotic lesions using a total RNA purification plus kit. Reverse transcription used SuperScript™ III First-Strand Synthesis System. Specific primer sets for mouse ICAM-1 (forward: AAACCAGACCCTGGAACTGCAC; reverse: GCCTGGCATTTCAGAGTCTGCT), VEGF (forward: CCTGAATCCTGGGAAATGTGCC; reverse: CGATTCGCACACGGTCTTCTGT), caspase-3 (forward: GGAGTCTGACTGGAAAGCCGAA; reverse: CTTCTGGCAAGCCATCTCCTCA), and GAPDH (forward: CATCACTGCCACCCAGAAGACTG; reverse: ATGCCAGTGAGCTTCCCGTTCAG) measured mRNA levels using PowerUp SYBR Green Master Mix (ThermoFisher Scientific) on a StepOneplus™ real-time PCR system. Relative gene expression was analyzed according to the 2^-ΔΔCt^ method. All samples were assayed in triplicate. Melting curve analysis determined the specificity of the amplified products and the absence of primer-dimer formation.

### Immunohistochemistry

The rehydrated paraffin sections were immersed in 3% H_2_O_2_ (in 100% methanol) for 10min to remove endogenous peroxidase activity followed by washing with PBS x3. Tissues were blocked with 0.5% BSA for 30min, then, incubated with monoclonal anti-mouse ICAM-1, VEGF, caspase-3 [1:2,000 (v/v)] or corresponding isotype IgG for 1h at room temperature. After washing, the slides were incubated with either anti-mouse or -rabbit secondary antibody for 40min at room temperature. The immunoreactivity was detected with 3,3’-diaminobenzidine (DAB) chromogen. Sections were lightly counterstained with hematoxylin for 1min and dehydrated in a gradient of alcohol and xylene. The slides were mounted with Permount mounting medium and examined with an Olympus BX43 light microscope. Immunoreactivity of different molecules were quantified using the Image-J v1.53 DIA software as previously described (24).

### Isobaric tagging for relative and absolute quantification gel-free proteomics

Total protein was extracted from plain culture medium and 3 batches of ADSC-CM for iTRAQ labeling. The samples (100 μg) were digested for 16 h at 37°C (protein:trypsin=30:1) using Trypsin Gold (Promega, Madison, WI, USA). Digestate was processed with 4-plex iTRAQ (Applied Biosystems, Waltham, MA, USA) labelling following the manufacturer’s instructions. Samples were labeled with the iTRAQ 114, 115, 116 and 117 (plain culture medium). Strong cation exchange (SCX) chromatography was performed using the LC-20AB HPLC Pump system (Shimadzu, Japan). SCX fractions were suspended in buffer A (2% ACN, 0.1% FA) followed by a centrifugation at 20,000x g for 10 min. Then, 10 μL of supernatant were loaded onto a 2-cm C18 trap column on a LC-20AD nanoHPLC (Shimadzu, Japan) by the auto sampler and eluted onto a 10-cm analytical C18 column (inner diameter 75 μm) packed in-house. Finally, the chromatographic conditions were restored in 1 min. Data acquisition was performed by a Q EXACTIVE (Thermo Fisher Scientific, CA) coupled to the HPLC. Compared to plain culture medium, proteins in ADSC-CM with 1.5-fold increase were selected for further analysis. All the differentially expressed proteins were first analyzed *via* UniProt (http://www.uniprot.org/). Further data mining was done using Ingenuity Pathway Analysis (IPA) software (Qiagen, Hilden, Germany).

## Results

### Characterization of ADSCs

**Figure 2:**
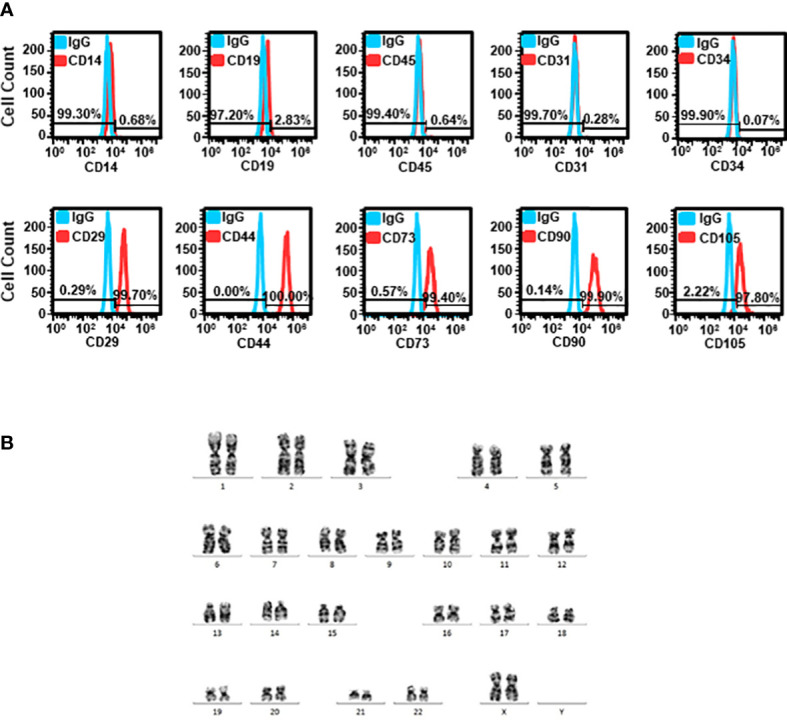
Characterization of ADSCs **(A)** Cells isolated from adipose tissue were stained with various markers, including CD29, CD44, CD73, CD90 and CD105 for MSC, CD14 for monocytes, CD19 for B-cells, CD31 for endothelial cells, CD34 for hematopoietic cells, and pan-leukocyte marker, CD45. Representative histograms of flow cytometric analysis are shown. **(B)** Representative karyotyping of cells isolated from adipose tissue.

The cells isolated from lipoaspiration-derived adipose tissue were subjected to flow cytometric analysis of various markers. The expression of CD14, CD19, CD45, CD31, and CD34 was absent in 99.70 ± 0.21%, 97.37 ± 1.13%, 99.57 ± 0.17%, 98.63 ± 0.64%, and 99.83 ± 0.03% of cells, respectively. The cells expressing CD29, CD73, CD90 and CD105 were 99.13 ± 0.30%, 98.20 ± 0.62%, 99.33 ± 0.35%, and 95.50 ± 1.69%, respectively (**Figure 2A**). A representative karyotyping showed that the adipose tissues were obtained from patients with normal karyotype (**Figure 2B**).

### ADSC-CM inhibits the development of endometriosis

**Figure 3:**
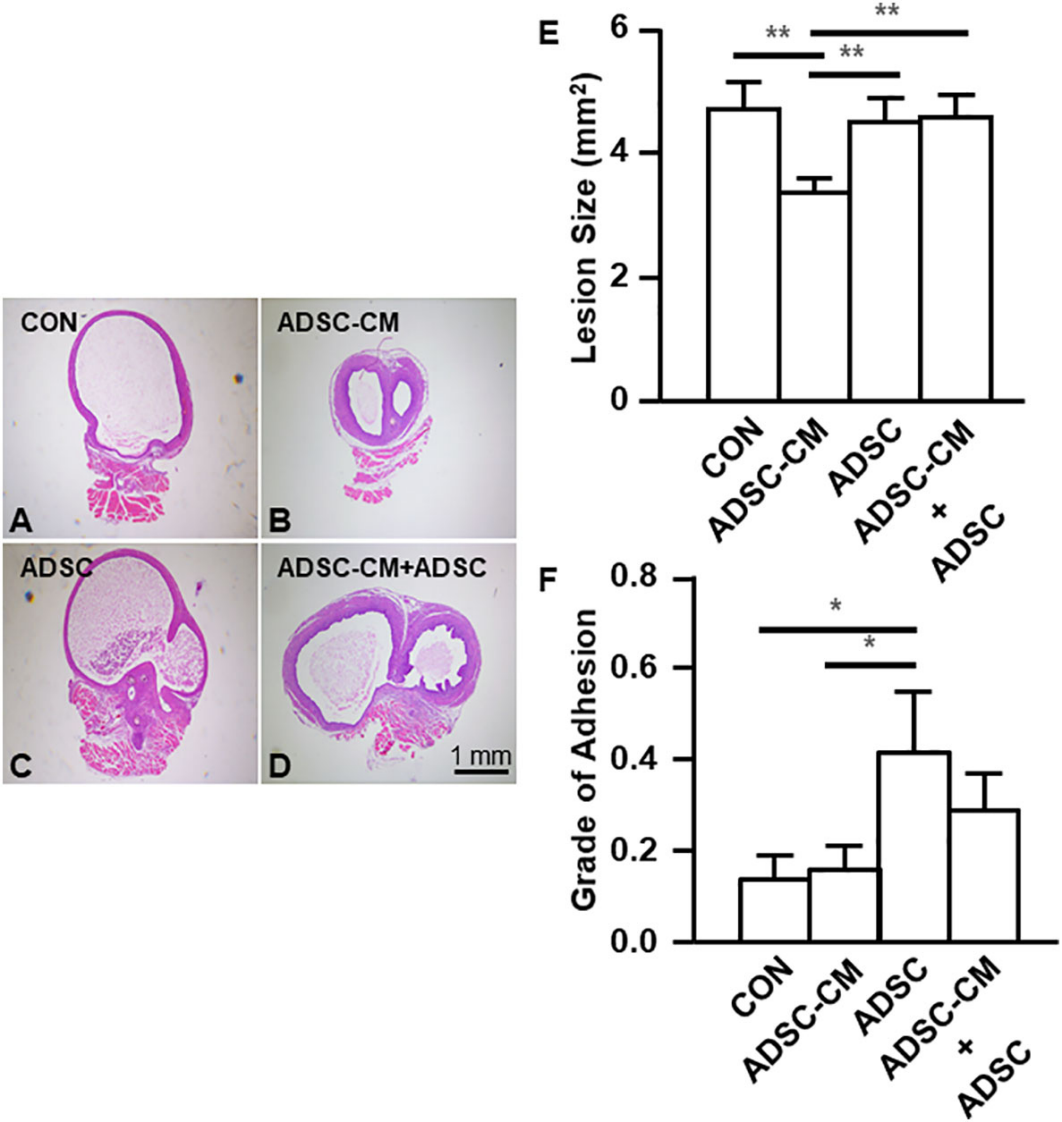
ADSC-CM inhibits the development of endometriosis Uterine tissue was sutured to the peritoneal wall of female C57B/6 mice and treated with **(A)** Culture medium, **(B)** ADSC-CM, **(C)** ADSC, **(D)** ADSC-CM + ADSC. **(E)** Lesion size and **(F)** grade of adhesion were measured. The data were reported as mean ± SEM. n=6; *p < 0.05.; **p < 0.01.

Endometriotic lesions were stained with hematoxylin and eosin. Compared with control (**Figure 3A**), ADSC-CM decreased the area of endometriotic lesions by 27.64 ± 3.02% (**Figures 3B, E**). The addition of ADSCs alone did not show inhibition on endometriotic lesions (**Figures 3C, E**). In contrast, the inhibitory effect of ADSC-CM was obliterated by adding ADSCs (**Figures 3D, E**). Although ADSC-CM treatment did not show improvement in peritoneal adhesion, the addition of ADSCs alone increased the peritoneal adhesion (**Figure 3F**).

### The expression of ICAM-1 and VEGF was suppressed by ADSC-CM

**Figure 4:**
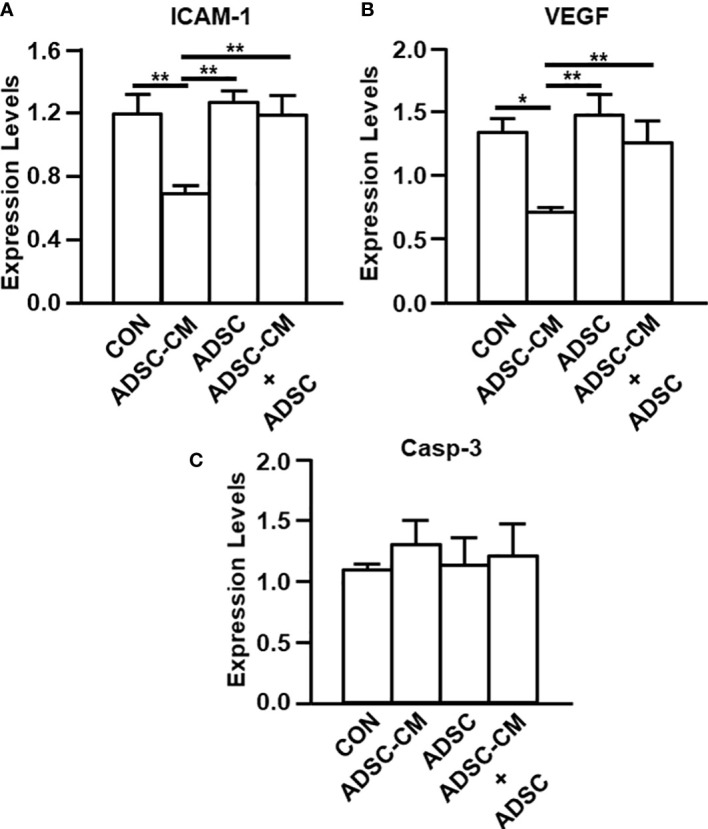
qRT-PCR of endometriosis-associated molecule expression The expression of **(A)** ICAM-1, **(B)** VEGF and **(C)** Caspase-3 in the endometriotic lesions were examined by qRT-PCR. The data were reported as mean ± SEM. n=6; *p < 0.05.; **p < 0.01.

Quantitative RT-PCR and IHC were used to evaluate the expression of ICAM-1 and VEGF in endometriotic lesions. Quantitative RT-PCR demonstrated that ICAM-1 expression was inhibited by ADSC-CM by 39.15 ± 6.99%, whereas the addition of ADSCs not only did not exhibit inhibition by itself, but also blocked the ADSC-CM-induced inhibition by 75.02 ± 18.76% (**Figure 4A**). Compared to control, ADSC-CM significantly suppressed VEGF mRNA expression by 44.93 ± 4.46%. The addition of ADSCs did not exert any effects on VEGF expression, compared with control. The down-regulation of VEGF mRNA by ADSC-CM was inhibited by the addition of ADSCs by 75.32 ± 23.24% (**Figure 4B**). The expression of caspase-3 was not affected by any treatment (**Figure 4C**).

**Figure 5:**
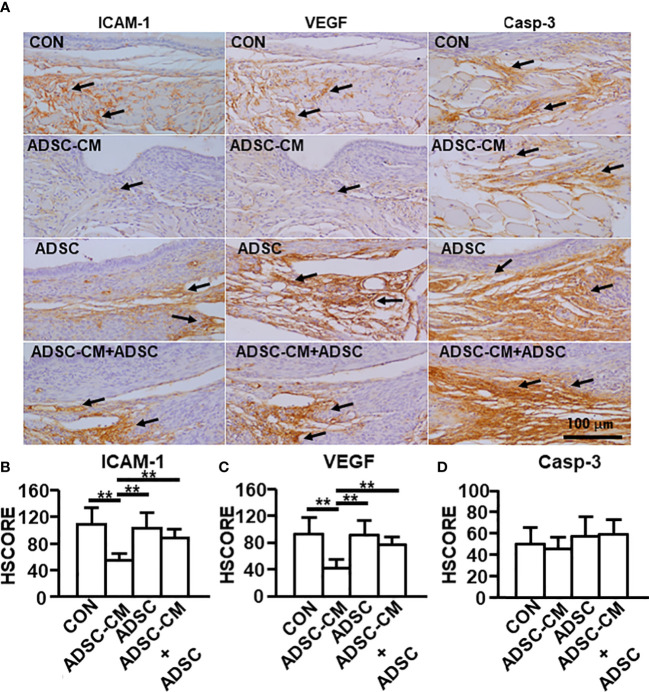
Immunohistochemical staining of endometriosis-associated molecules The expression of **(A)** ICAM-1, VEGF and caspase-3 in endometriotic lesions was demonstrated by immunohistochemistry. The immunoreactivity of **(B)** ICAM-1, **(C)** VEGF and **(D)** Caspase-3 in the tissue were semi-quantified by HSCORE. The data were reported as mean ± SEM. n=6; **p < 0.01.

Consistently, immunostaining revealed that ADSC-CM reduced ICAM-1 and VEGF expression by 43.74 ± 11.36% and 44.59 ± 16.84%, respectively. The existence of ADSCs did not show effect on the expression of ICAM-1 (**Figures 5A, B**) and VEGF (**Figures 5A, C**), however, suppressed the inhibitory effect of ADSC-CM on the expression of ICAM-1 (**Figures 5A, B**) and VEGF (**Figures 5A, C**) by 68.52 ± 10.33% and 47.71 ± 12.81%, respectively. Caspase-3 expression in the endometriotic tissue was regulated by neither ADSC-CM nor ADSCs (**Figures 5A, D**).

### ADSC-CM improved pregnancy outcomes in mice with endometriosis

**Figure 6:**
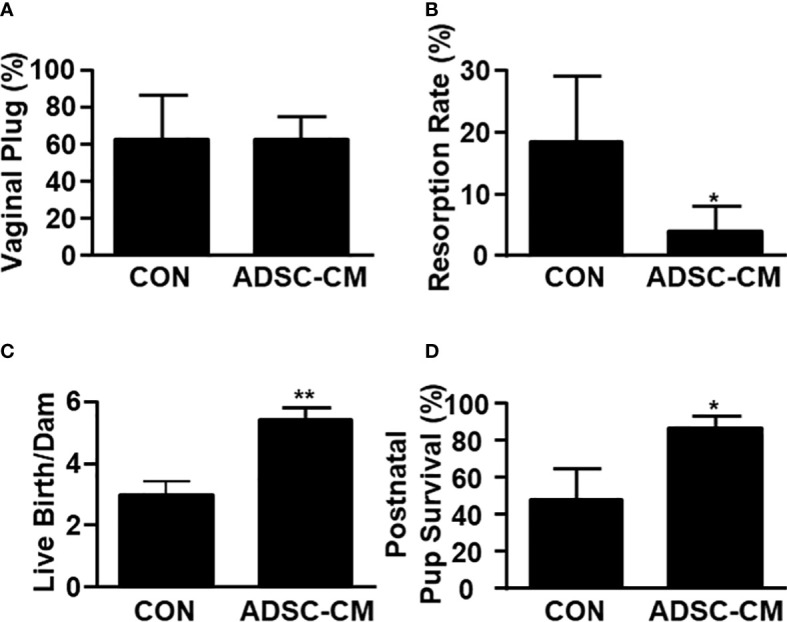
ADSC-CM Improves Pregnancy Outcomes in Mice with Endometriosis Fecundity and pregnancy outcomes represented by **(A)** numbers of vaginal plug, however, **(B)** resorption rate, **(C)** live births per dam, **(D)** pup survival rate at 1 week-old were recorded in mice complicated with endometriosis treated with or without ADSC-CM. The data were reported as mean ± SEM. n=4; *p < 0.05.; **p < 0.01.

Mice were mated with male mice at day 28 and allowed to proceed to delivery. The number of vaginal plug showed no different between control and ADSC-CM-treated groups (**Figure 6A**). The resorption rate was reduced by ADSC-CM from 18.33 ± 10.67% to 4.00 ± 4.00% (**Figure 6B**). The number of live birth/dam was increased by ADSC-CM treatment from 3.00 ± 0.41 to 5.40 ± 0.40 in mice with endometriosis (**Figure 6C**). The survival rate of pup at 1 week-old was improved from 47.92 ± 16.80% to 86.67 ± 6.24% (**Figure 6D**).

### ADSC-CM improved endometrial receptivity in mice with endometriosis

**Figure 7:**
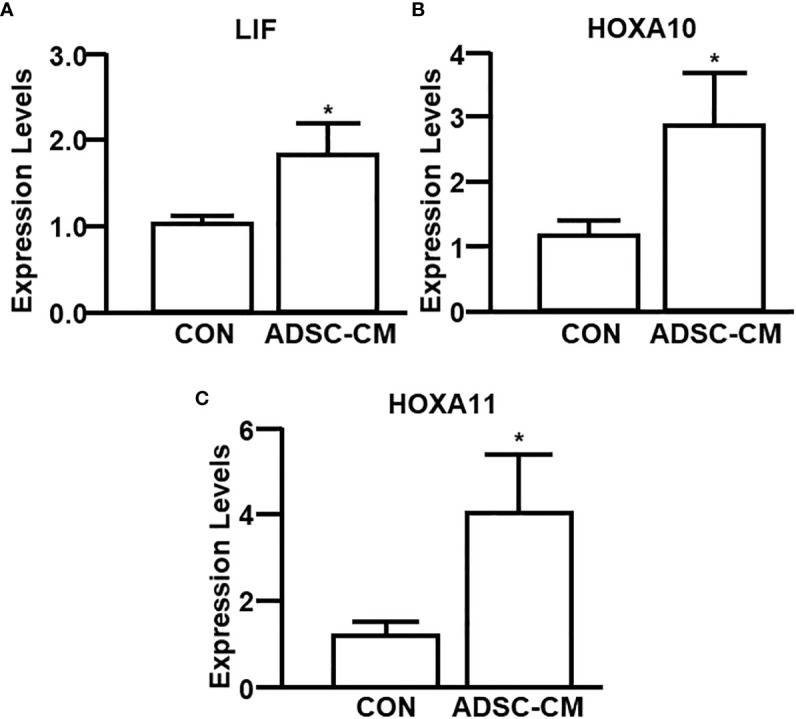
qRT-PCR of receptivity markers The expression of **(A)** LIF, **(B)** HOXA10 and **(C)** HOXA11 in the eutopic endometrium of mice with endometriosis at GD4 was examined by qRT-PCR. The data were reported as mean ± SEM. n=6; *p < 0.05.

**Figure 8:**
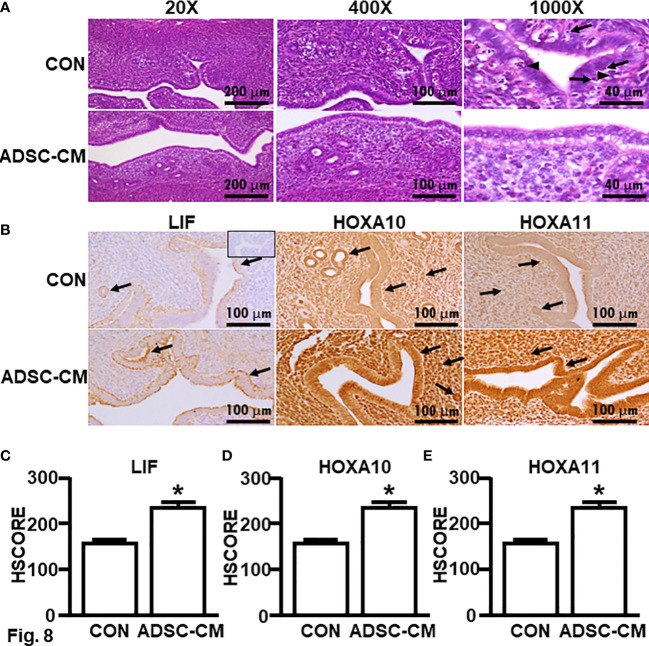
Histology and immunochemical staining of receptivity markers Eutopic endometria of mice with endometriosis at GD4 were subjected to **(A)** H & E stain. Arrows: lymphocytes; Arrow heads: polymorphonuclear cells. **(B)** Immunohistochemistry was used to assess the expression of **(C)** LIF, **(D)** HOXA10 and **(E)** HOXA11. The data were reported as mean ± SEM. n=6; *p < 0.05.

To further study the mechanism associated with enhanced pregnancy outcomes, the endometrial receptivity of eutopic endometrium of mice with endometriosis was examined. **Figure 7** demonstrated ADSC-CM up-regulated the mRNA expression of receptivity markers, including LIF, HOXA10 and HOXA11, by 1.72 ± 0.50- (**Figure 7A**), 3.25 ± 0.76- (**Figure 7B**), 4.67 ± 1.74-fold (**Figure 7C**), respectively. Compared to the control eutopic endometrium, ADSC-CM eliminated the immune cell infiltration, including lymphocytes and polymorphonuclear cells, in the sub-epithelial regions in eutopic endometrium of pregnant endometriotic mice at GD 4.5 (**Figure 8A**). IHC revealed that ADSC-CM consistently promoted the expression of LIF (**Figures 8B, C**), HOXA10 (**Figures 8B, D**) and HOXA11 (**Figures 8B, E**) by 90.17 ± 9.32%, 83.14 ± 5.95%, and 53.84 ± 9.46%, in epithelium, stroma and glands.

### Proteomics analysis of ADSC-CM

ADSC-CM was subjected to iTRAQ analysis. A total of 1,348 proteins were detected in ADSC-CM (**Supplementary Table 1**). The results were further analyzed with Ingenuity Pathway Analysis (IPA) software. These proteins were shown to be involved in the signaling of 598 canonical pathways (**Supplementary Table 2**). Among which, acute phase response signaling, LXR/RXR activation, BAG2 signaling pathway, FAT10 signaling pathway, and clathrin-mediated endocytosis signaling were the most significant pathways associated with ADSC-secreted proteins. These ADSC-derived proteins also played a role in 500 diseases and biofunctions (**Supplementary Table 3**) as well as 746 Tox functions (**Supplementary Table 4**). Specifically, 82 molecules were involved in the pathogenesis of endometriosis. A total of 4,033 upstream regulators were involved in the regulation of the expression of proteins in ADSC-CM (**Supplementary Table 5**). This regulation acts through 2,986 casual networks (**Supplementary Table 6**). The proteins in ADSC-CM form 25 interactive networks (**Supplementary Table 7**). Among 1,348 proteins, pentraxin 3 (PTX3) was found to interact with 114 molecules (**Supplementary Table 8**), particularly involved in regulating a variety of inflammatory cytokines and their receptors, including CCL2, CCL4, CCL5, CCL7, CCL8,CXCL8, CCR2, CCR5, and CX3CR1. In addition, IL-1α, IL-1β, TNF-α, IL-10, TLR2, TLR3, and TLR4 were shown to up-regulate PTX3, whereas PTX3 was down-regulated by IL-17A and TGF-β1.

## Discussion

Endometriosis is known to be an inflammatory disease of pelvic cavity. The treatment of endometriosis aims to not only eradicate the ectopic endometrium, but also reduce inflammation and its associated complications, such as pain and adhesion-related sequelae. Due to high recurrence rate of endometriosis after medical treatment, patients suffering from endometriosis are usually destined for either infertility or surgical removal of uterus and adnexae after completing childbearing. Thus, an effective treatment to improve fertility and ameliorate symptoms is required to promote patients’ quality of lives. ADSC-CM was used to treat endometriosis in the current study. After isolation, the purity of ADSCs was verified by the absence of CD14 (monocyte), CD19 (B-cell), CD45 (pan-leukocyte), CD31 (endothelial cell), and CD34 (hematopoietic cell) expression accompanied by expressing CD29, CD44, CD73, CD90 and CD105 with normal karyotype. The pharmaceutical quality of media used for ADSC culture was validated by growth promotion test. The preparation of ADSC and ADSC-CM was under the guidance of GTP and GMP regulations. The ADSC-CM was shown to be free of bacterial endotoxin and mycoplasma in ensuring the sterility and safety of the products.

The experimental mice were treated with or without ADSCs and/or ADSC-CM at the beginning of the experiments, the reduction of lesion size by ADSC-CM indicates its inhibitory effect on newly formed endometriotic lesions. Although MSCs acquired from other tissues were demonstrated to be anti-inflammatory (25), in the current study, the addition of ADSCs appears to offset the inhibition by ADSC-CM. Whether it’s because of a tissue-specific effect or dosage-related issue needs to be further scrutinized. Also, treatment with ADSCs alone did not increase the growth of endometriotic tissue, suggesting that stem cells were not involved in the growth of endometriosis under the pathogenic microenvironment. Furthermore, to test its therapeutic effect on the pre-existing endometriotic lesions in the future studies, the animals will not be treated until day 21 when the endometriotic lesions develop. Endometriosis is an inflammatory disease which usually leads to severe pelvic adhesion. In the current model, no apparently improvement in adhesion by ADSC-CM was observed. Although not statistically significant in comparison to non-treated group (p=0.574) and ADSC-CM-treated group (p=0.68), the addition of ADSCs seems to aggravate the adhesion. In addition to ADSC-CM, further studies are required to elucidate whether CM derived from stem cells isolated from mesenchyme of other tissues also exhibits similar inhibitory effects on the development of endometriosis.

Endometriotic patients exert reduced cycle fecundity, suggesting a decrease in receptivity of the eutopic endometrium (26). Defective endometrial receptivity can lead to adverse pregnancy outcomes, such as miscarriage, preeclampsia, and fetal growth restriction, potentially due to suboptimal trophoblast invasion (27). Therefore, in addition to alleviating the lesions and their associated symptoms, improving eutopic endometrial receptivity is critical in treating endometriotic patients who desire reproduction. In the current study, the percentages of the existence of vaginal plug after mating are similar between ADSC-CM-treated and control endometriotic mice, indicating the mating behavior of mice was not changed by ADSC-CM. However, the reduction of resorption rate as well as the improvement of live birth number and postnatal pup survival rate by ADSC-CM suggest the enhancement of endometrial receptivity by ADSC-CM in endometriotic mice.

**Figure 9:**
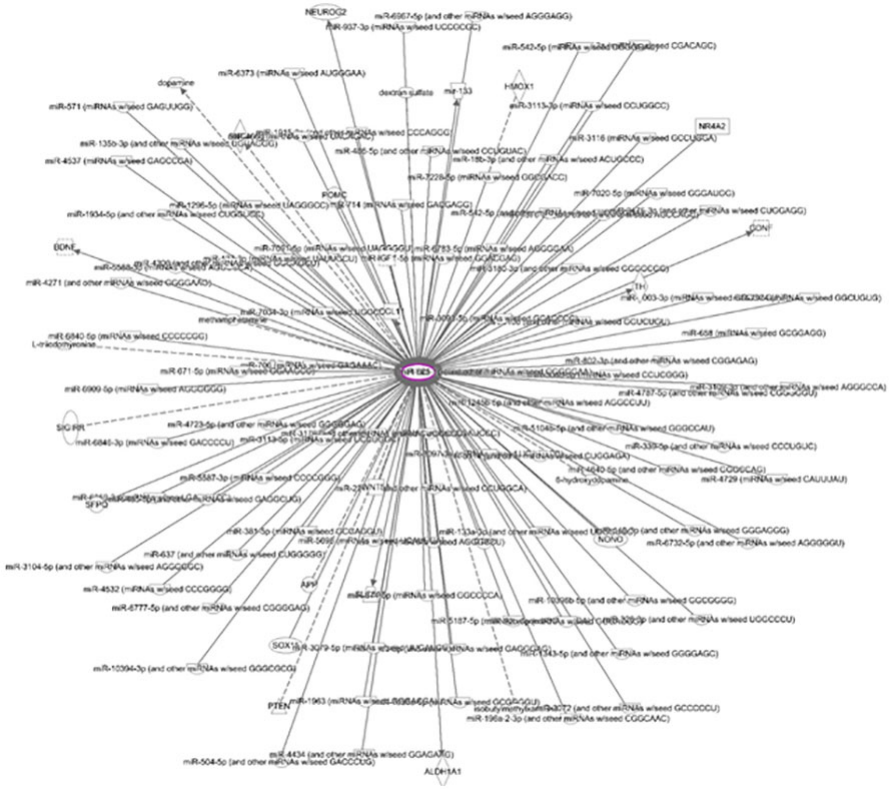
Network of molecules interacting with PTX3 One hundred and fourteen molecules interact with PTX3 in the ADSC-CM.

Although an array of secreted proteins, including an extensive range of cytokines, chemokines, adhesion molecules, proteases, shed receptors, and growth factors, have been shown in several studies, a complete secretome profile has not been demonstrated (28). In our preliminary analysis, PTX3 appears to be a potential candidate crucial for the inhibition of endometriosis by ADSC-CM (**Figure 9**). PTX3 functions as a soluble pattern recognition receptor (29). In human endometrial stromal cells, estradiol and progesterone induce PTX3 expression that plays an important role in implantation of blastocyst (30). Through binding with heavy chain-hyaluronan (HC-HA) (31), HC-HA PTX3 exerts anti-angiogenic (32), anti-inflammatory, anti-scarring (33) effects. Such binding also stabilizes HC-HA in the cumulus oophorous complex matrix (34). Macrophages are polarized to M2 subtype by HC-HA PTX3 (35). Nevertheless, the aforementioned proteins, DNA fragments, lipids, and RNA species (microRNA and long non-coding RNAs) can also be secreted *via* membrane-encapsulated particles, namely extracellular vesicles (EVs). Thus, the inhibition of the development of endometriosis by ADSC-CM is more likely to be attributed to a combinatory effect of these secreted molecules. The proteomic analysis in this study reveals the protein profile of ADSC-CM. Additional studies are required to demonstrate the distribution of various protein and non-protein molecules.

In conclusion, the current study showed for the first time that ADSC-CM effectively reduces the development of endometriosis and improves pregnancy outcomes. These findings open a new avenue in establishing a novel therapeutic strategy to combat endometriosis. These results can potentially be translated to clinical management of endometriosis.

## Data availability statement

The original contributions presented in the study are included in the article/**Supplementary Material**. Further inquiries can be directed to the corresponding author.

## Ethics statement

The studies involving human participants were reviewed and approved by E-Da Hospital Institutional Review Boards. The patients/participants provided their written informed consent to participate in this study. The animal study was reviewed and approved by E-Da Hospital Institutional Animal Care and Use Committee.

## Author contributions

SJH and L-YS designed research and drafted the manuscript. Y-PH and C-CC provided the technical assistance and consultation for experimental design. C-YH and Y-CY performed animal studies, qRT-PCR, IHC, and data analyses. Y-HH provided the adipose tissue from lipoaspiration. L-YS and J-CL isolated the ADSCs and did the quality control tests. J-HC performed the proteomics analysis of ADSC-CM. SH did the IPA analysis of proteomics data. All authors contributed to the article and approved the submitted version.
